# CCR2 Acts as Scavenger for CCL2 during Monocyte Chemotaxis

**DOI:** 10.1371/journal.pone.0037208

**Published:** 2012-05-17

**Authors:** Silvia Volpe, Elisabetta Cameroni, Barbara Moepps, Sylvia Thelen, Tiziana Apuzzo, Marcus Thelen

**Affiliations:** 1 Institute for Research in Biomedicine, Bellinzona, Switzerland; 2 Institute of Pharmacology and Toxicology, University of Ulm Medical Center, Ulm, Germany; University of Leuven, Rega Institute, Belgium

## Abstract

**Background:**

Leukocyte migration is essential for effective host defense against invading pathogens and during immune homeostasis. A hallmark of the regulation of this process is the presentation of chemokines in gradients stimulating leukocyte chemotaxis via cognate chemokine receptors. For efficient migration, receptor responsiveness must be maintained whilst the cells crawl on cell surfaces or on matrices along the attracting gradient towards increasing concentrations of agonist. On the other hand agonist-induced desensitization and internalization is a general paradigm for chemokine receptors which is inconsistent with the prolonged migratory capacity.

**Methodology/Principal Findings:**

Chemotaxis of monocytes was monitored in response to fluorescent CCL2-mCherry by time-lapse video microscopy. Uptake of the fluorescent agonist was used as indirect measure to follow the endogenous receptor CCR2 expressed on primary human monocytes. During chemotaxis CCL2-mCherry becomes endocytosed as cargo of CCR2, however, the internalization of CCR2 is not accompanied by reduced responsiveness of the cells due to desensitization.

**Conclusions/Significance:**

During chemotaxis CCR2 expressed on monocytes internalizes with the bound chemoattractant, but cycles rapidly back to the plasma membrane to maintain high responsiveness. Moreover, following relocation of the source of attractant, monocytes can rapidly reverse their polarization axis organizing a new leading edge along the newly formed gradient, suggesting a uniform distribution of highly receptive CCR2 on the plasma membrane. The present observations further indicate that during chemotaxis CCR2 acts as scavenger consuming the chemokine forming the attracting cue.

## Introduction

Leukocyte migration is essential for innate and adaptive immune responses and is mainly orchestrated by chemokines presented in chemotactic gradients generated by target tissues [1], [2]. Binding of chemokines to their cognate chemokine receptors expressed on leukocytes induces directional migration. Chemokine receptors belong to the rhodopsin family of heterotrimeric G-protein-coupled receptors (GPCRs) and elicit intracellular signals via pertussis toxin sensitive G_i_-proteins [3]. Signal amplitude and duration depend on ligand concentration and are regulated through receptor desensitization, phosphorylation and internalization. Most of these responses require efficient coupling to G_i_-proteins and the activation of downstream signaling pathways. Second messenger-dependent kinases, such as protein Kinase A or C, usually desensitize receptors through transient phosphorylation [4]–[6]. By contrast, GPCR kinase-mediated phosphorylation can be pertussis toxin insensitive and leads to arrestin binding and receptor internalization [7], [8], which typically proceeds through clathrin coated pits. However, in some cases chemokine receptor internalization can also involve caveolin leading to the formation of caveosomes, which then fuse with early endosomes, joining the route of clathrin-dependent endocytosis [7], [9]–[12]. Internalized chemokine receptors residing on early endosomes can be directed to recycling pathways or remain on the organelles that are processed to late endosomes and ultimately to lysosomes where protein degradation occurs [7].

CCR2 is an inflammatory chemokine receptor which mediates migration of lymphocytes, neutrophils, dendritic cells and monocytes/macrophages [2], [13]. Several CC-chemokines bind to human CCR2 and stimulate cell migration, such as CCL2, CCL7, and CCL13 [14]–[16]. Typically CCR2-triggred responses are sensitive to pertussis toxin treatment, such as calcium mobilization and chemotaxis [17], [18]. Bulk treatment of monocytes with CCL2 leads to phosphorylation of the C-terminus of CCR2 and to its rapid internalization via arrestin binding and a combination of clathrin coated pits and caveosomes [11], [19]. Internalized CCR2 can be recycled to the membrane [20] or become down regulated through lysosomal degradation [7], [21]. Interestingly, in the case of CCR2 receptor internalization can be dissociated from functional responses. In line with this it was shown using phosphorylation-deficient mutants that CCR2 internalization is not essential for CCL2-stimulated chemotaxis [22].

Receptor localization during cell migration appears to be cell context dependent. It was reported that in polarized lymphocytes chemotaxis-mediating receptors accumulate at the leading edge [23], although this conclusion was questioned when analyzed in transfected leukemia PBL-985 cells [24]. We have previously shown that in transfected monocytic THP-1 cells chemotaxis-mediating receptors remain uniformly distributed over the entire cell perimeter [25]. In addition, using time-lapse video microscopy we showed that migrating monocytes rapidly flip their polarization axis when the source of attractant was relocated to the opposite site (rear) of the cells. The rapid adaptation to the altered location of the chemoattractant suggests that receptors remain evenly distributed on the surface of monocytes to allow fast responsiveness in any direction rather than concentrating at the leading edge [25].

In the present study we addressed the question whether endogenous chemokine receptors internalize during migration. To visualize the dynamics of chemokine receptor localization in primary untransfected human monocytes we used fluorescent protein-tagged CCL2 to stimulate chemotaxis via endogenous CCR2. Using time-lapse video microscopy we show that CCL2 is efficiently and selectively internalized during migration by a CCR2 mediated process. Furthermore sustained chemokine uptake during migration is consistent with the view that CCR2 rapidly cycles from the plasma membrane to endosomal structures deploying extracellular chemoattractant for degradation. Our observations also indicate that chemokine receptors consume the attractant from the surrounding medium during migration potentially reducing the strength of the cue.

## Materials and Methods

### DNA constructs

Human CCL2 obtained by PCR from plasmid pBluescript-CCL2 using primers 5′-A AAA TCT AGA GCC ACC ATG AAA GTC TCT GCC GCC CTT C-3′ and 5′-A AAA CTC GAG AGT CTT CGG AGT TTG GGT TTG C-3′ was subcloned in frame with the red fluorescent protein mCherry [26] preceded by a short linker sequence (coding for amino acids: SGGGGSGGGGSGGGGS) which was fused to the fluorescent protein via EcoR1 site removing the start methionine. The construct was excised by enzymatic restriction with Xba1 (5′) and Kpn1 (3′), the Kpn1 site was blunted with T4 polymerase (NewEngland Biolabs) according to the manufacturer instructions and the product ligated into Xba1 and Sma1 restriction sites of pVL1392 (BD Biosciences) yielding pVL1392-CCL2-mCherry. Baculoviruses were raised in Sf9 cells (Invitrogen, B825-01) using the BaculoGold™ kit (BD Biosciences) according to the manufacturer's instructions and used to infect High Five cells to express the recombinant protein.

Human CCL20 cDNA was prepared from PMA-stimulated GM-1 cells [27] using a standard RT-PCR reaction. Briefly GM-1 cells were stimulated with 50 ng/ml PMA for 8 h, harvested and mRNA was extracted using Trizol (Invitrogen) according to the manufacturer's instructions. Reverse transcription of 5 µg mRNA was performed with SuperScript® VILO™ cDNA Synthesis kit (Invitrogen 11754-050). The primers 5′-A AAA TCT AGA ***GCC ACC*** ATG TGC TGT ACC AAG AGT TTG C-3′ and 5′-AAC AAA CTC GAG CAT GTT CTT GAC TTT TTT ACT GAG G-3′ were used to amplify the CCL20 coding sequence (including the signal peptide). The PCR product was restricted with XbaI and XhoI and replaced for the CCL2 sequence in pVL11392-CCL2-mCherry. Similarly the sequence of mCherry was replaced with the sequence of Venus (EYFP) which was obtained by PCR using a Venus fusion protein as template [25]. The sequence integrity of all constructs was verified by DNA sequencing.

### Purification of tagged chemokines

Baculovirus culture supernatants were filtered through 0.2 µm Stericups (Invitrogen) to remove cell debris. After the supernatants were adjusted to pH 7.3, S Sepharose (GE Healthcare) was added and the suspension incubated for 3 h at 4°C under continuous agitation. S Sepharose was collected by centrifugation, washed with 50 mM NaCl, 20 mM NaP_i_ pH 7.3 and bound chemokines (visible by color) were eluted with a 1 M NaCl in a minimal volume. Eluted proteins were subjected to gel filtration (Superdex 200 PC 30/3.2, GE Healthcare). Peak fractions containing pure (>90%) chemokine, determined by calculating the absorbance ratio of the fluorescent protein in the visible light and at 280 nm, were pooled and stored at −80°C.

### Cell isolation

PBMC were isolated from heparinized venous blood of healthy volunteers by Ficoll-Paque™ Plus (Ge-Healthcare) density gradient centrifugation as recommended by the manufacturer. Monocytes were isolated from PBMC by MACS with the Monocyte Isolation Kit II (Miltenyi) and their purity was determined by FACS by staining the cells with anti-CD14 and anti-CD45. Isolated Monocytes were resuspended at 0.5×10^6^/ml in RPMI 1640 supplemented with 10% fetal bovine serum (FBS, HyClone), 1% glutamax, 50 U/ml of penicillin and 50 µg/ml of streptomycin (all Invitrogen). When indicated, cells were treated with 100 nM 17-hydroxy-wortmannin (HWT) or with 100 nM of the CCR2 antagonist BMS CCR2 22 (TOCRIS Bioscience) for 30 min and assayed in the presence of the compound. Monocytes were treated with 2 µg/ml *Bordetella Pertussis* toxin (List Biological Laboratories) washed and subjected to video microscopy.

### Calcium and Chemotaxis

Intracellular free calcium in monocytes was measured as previously described [25]. Chemotaxis assays were performed in triplicate in 48-well Boyden chambers (NeuroProbe, Gaithersburg, MD), using 5 µm pore-sized polyvinylpyrrolidone-free polycarbonate membranes. Chemotaxis medium (RPMI-1640 supplemented with 1% FBS, 1% glutamax, Pen/Strep) containing chemoattractants was added to the lower wells. Freshly isolated monocytes (10^5^ per well) in chemotaxis medium were added to the upper wells and incubated for 60 min at 37°C in 5% CO_2_ atmosphere. Cells were removed from the upper part of the membrane with a rubber policeman. Monocytes attached to the lower side of the membrane were fixed and stained. Migrated cells were counted in five randomly selected fields of 100-fold magnification.

### Microscopy

Laser scanning confocal microscopy was performed with a Leica DI6000 microscope stand connected to a SP5 scan head equipped with a temperature controlled chamber. If not indicated separately videos were routinely recorded with a 63× objective. Live cell cultures were placed in a humidified air/CO_2_-controlled incubator which was mounted on the microscope stage. For time-lapse video microscopy cells (0.5×10^6^/ml) were resuspended in D-PBS containing Ca^2+^and Mg^2+^ (Invitrogen) supplemented with 1% FBS, 1% Pen/Strep, 0.04 mM sodium pyruvate, 1 mg/ml fatty acid free BSA (Sigma), 1 mg/ml glucose. Cells were plated on glass bottom petridishes (MatTek cultureware) coated with D-polylysine (5 µg/ml) and subsequently overlaid with a 1∶80 diluted MatrigelH (BD Biosciences) solution at 4°C for 30 min.

Chemoattractants were dispersed with a micropipette (FemtotipII, Eppendorf) controlled by micromanipulator (Eppendorf) at a constant backpressure of 15 hPa (Femtojet, Eppendorf).

## Results

### Responsiveness of CCR2 to CCL2 and CCL2mCherry

**Figure 1:**
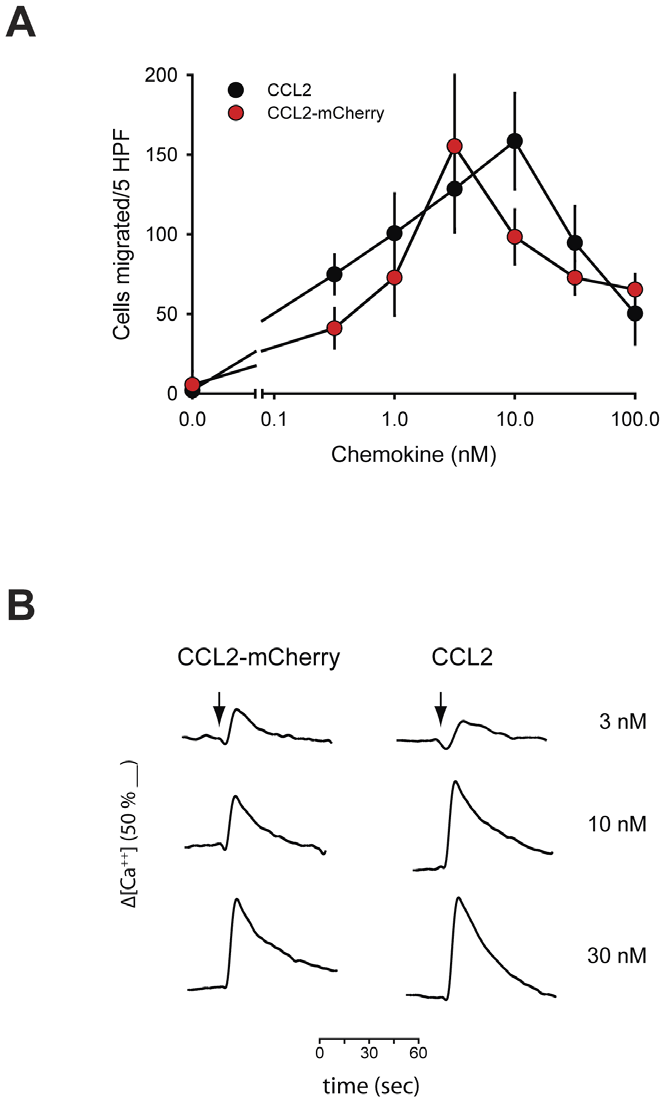
CCL2-mCherry has similar potency as wild type CCL2. Freshly isolated human monocytes chemotaxis was measured in Boyden chamber chemotaxis assays at the indicated concentrations (A). Wild type CCL2 (black circles) and CCL2-mCherry (red circles) was added to the lower compartment at the indicated concentrations. Samples from atypical experiment were determined in triplicates. Intracellular free calcium in fura-2 loaded monocytes stimulated with CCL2-mCherry (left traces) or wild type CCL2 (right traces). Both chemokines gave similar responses at the indicated concentrations (right). Individual traces from a typical experiment performed in duplicates.

To monitor receptor ligand interactions in primary non-transfected monocytes we generated a fluorescent analogue of CCL2 by fusing the monomeric red fluorescent protein mCherry to its C-terminus (CCL2-mCherry) [28]. Given the relative size of the fluorescent protein moiety with respect to the chemokine we first compared the efficiency of the fusion protein and wild type counterpart in chemotaxis and calcium mobilization assays. Figure 1 demonstrates that CCL2 and CCL2-mCherry possess similar efficacy and efficiency in eliciting migration and calcium fluxes in human monocytes. Chemotaxis was measured in Boyden chamber assays [25] and showed the typical bell shaped dose-response curve. Similarly, the stimulus-induced rise in intracellular free calcium ([Ca^2+^]_i_) was similar with both forms of CCL2. The results indicate that the fusion protein was equally potent as wild type CCL2, suggesting that attachment of mCherry does not interfere with receptor ligand interactions.

### Human monocyte migration towards CCL2-mCherry

**Figure 2:**
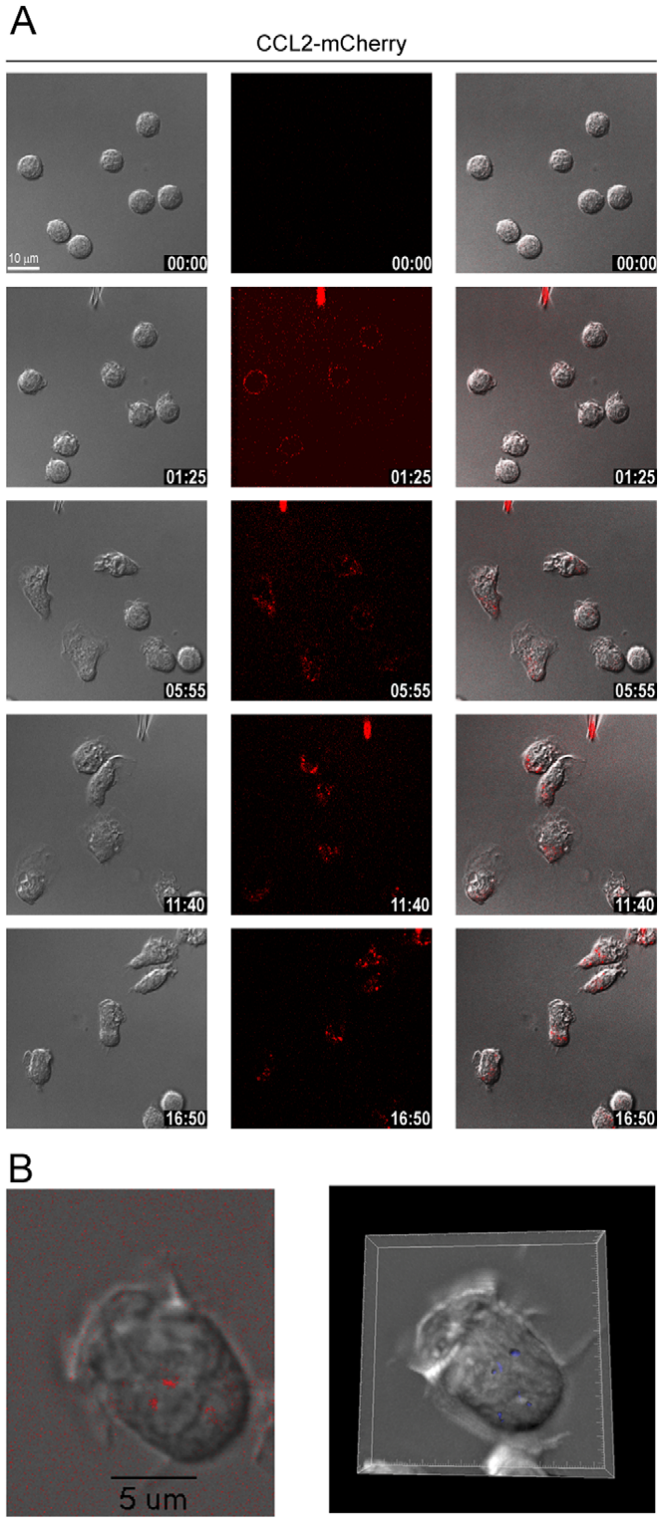
Monocytes internalize CCL2-mCherry during migration. Selected frames from time-lapse video microscopy (Movie S1) of migrating human monocytes recorded at 37°C (A). The micropipette was dispending 100 nM CCL2-mCherry with constant backpressure. Left panels phase images, middle panels CCL2-mCherry (ex 594 nm/em 620–680 nm), and right overlay. Time stamps are indicated in mm∶ss, calibration bar 10 µm (top left panel). (B) Single cell recorded at higher magnification (100×). Left plane from a confocal stack, right 3D rendering from the stack. The endosomal surface (blue) was calculated from the mCherry fluorescence using the Imaris software package (see also Movie S2).

Leukocytes exposed to chemokine gradients rapidly polarize forming a distinct leading edge and an uropod. The receptors which mediate the process remain uniformly distributed over the entire plasma membrane, but downstream signaling is polarized leading to the activation of PI 3-kinase exclusively at the leading edge [25]. Bulk-stimulation of leukocytes *in vitro* typically causes the desensitization and subsequent internalization of the chemoattractant receptors. We asked whether cells crawling along a chemotactic gradient remain responsive to the attractant over time allowing the continuous movement. To this end freshly isolated human monocytes were plated on glass-bottom dishes, coated with poly-D-lysine and a monolayer of matrigel [25], and placed in a temperature controlled ambience fitted on a confocal microscope stage. The attractant CCL2-mCherry was dispensed from a micropipette with constant pressure. Time-lapse video microscopy was recorded in the respective fluorescence channel and by phase contrast. Figure 2A and in more detail movie S1 demonstrate that human monocytes quickly orient toward the source of the attractant. Ligand association over the entire cell perimeter is observed after one minute and is followed by uptake as visualized by the localization of mCherry fluorescence in small endosomal-like structures. Figure 2B depicts a single plane of a confocal stack taken at high magnification (100×) of a monocyte migrating towards CCL2-mCherry. The right panel and movie S2 display the 3D computer-assisted reconstruction of the stacks confirming that mCherry is contained in intracellular structures (blue). Migration of monocytes towards the dispensing pipette was observed over a wide range of chemokine concentrations and continued for prolonged time even at elevated concentrations (10 µM, see below). However, dispensing an irrelevant chemokine or medium alone did not cause polarization of the cells (not shown).

Over time the endosomes become larger and accumulate preferentially at the rear of the cells, opposite to the source of the attractant. It is conceivable that initial small endosomes fuse to develop the larger structures, which appear with stronger fluorescence due to their enriched chemokine content (Fig. 2A).

Next, monocytes were allowed to migrate for 15 min towards CCL2 and then the dispensing pipette was removed from the medium (movie S3). After 30 min the cells had degraded all internalized CCL2-mCherry, lost polarity completely and returned to a spherical shape similarly to the appearance of resting cells. Nevertheless, upon reinsertion of the dispensing pipette monocytes rapidly polarized again and migrated toward the relocated source of chemoattractant avidly taking up CCL2-mCherry. The observation indicates that CCR2 remains responsive at the cell surface over the entire recording time, including after removal of the chemotactic cue.

### CCL2 uptake is CCR-mediated

**Figure 3:**
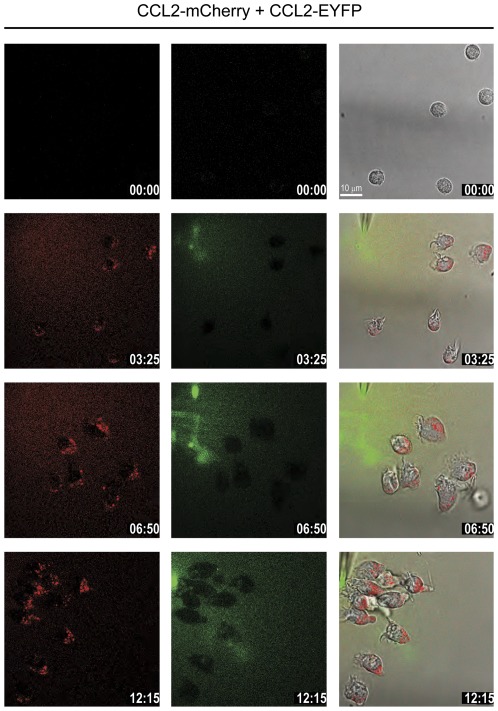
CCL2-mCherry uptake is not due to pinocytosis. Selected frames from time-lapse video microscopy (Movie S4) of migrating monocytes recorded at 37°C. The micropipette was dispending 1 µM CCL2-mCherry and 1 µM CCL20-EYFP with constant backpressure. Left panels CCL2-mCherry (ex 594 nm/em 620–680 nm), middle panels CCL20-EYFP (ex 488 nm/em 500–520 nm), and right panels merged frames overlaid with the corresponding phase image. Time stamps are indicated in mm∶ss.

The continuous migration toward the dispensing pipette and the concomitant intracellular accumulation of the chemoattractant is consistent with an active process of ligand uptake during chemotaxis. Three possible mechanism of chemokine internalization were considered: i) uptake by the cognate chemokine receptor CCR2, ii) uptake through the scavenger receptor D6 which is expressed on monocytes [29] and iii) internalization via (micro) pinocytosis. First we investigated the possibility of receptor-independent uptake by pinocytosis. To this end we produced recombinant human CCL20 fused to green fluorescent protein (CCL20-EYFP). CCL20 is a selective ligand for CCR6, which is expressed on lymphocytes and some dendritic cells, but not on monocytes [2]. Accordingly, neither CCL20 nor CCL20-EYFP induced chemotaxis of monocytes (not shown). When the dispensing pipette was filled with equal molar solutions of the green fluorescent CCL20 and the red fluorescent CCL2, monocytes migrated towards the source, but internalized exclusively the red fluorescent CCL2 (Fig. 3 and movie S4). The movie S4 shows the exclusive formation of red fluorescent endosomes even in close vicinity of the orifice of the pipette where the cells are surrounded by the green fluorescent CCL20. The images demonstrate an efficient capacity of the monocytes to discriminate between the two chemokines. The observation strongly argues against a receptor-independent, random fluid uptake, excluding pinocytosis as responsible mechanism of chemokine internalization.

**Figure 4:**
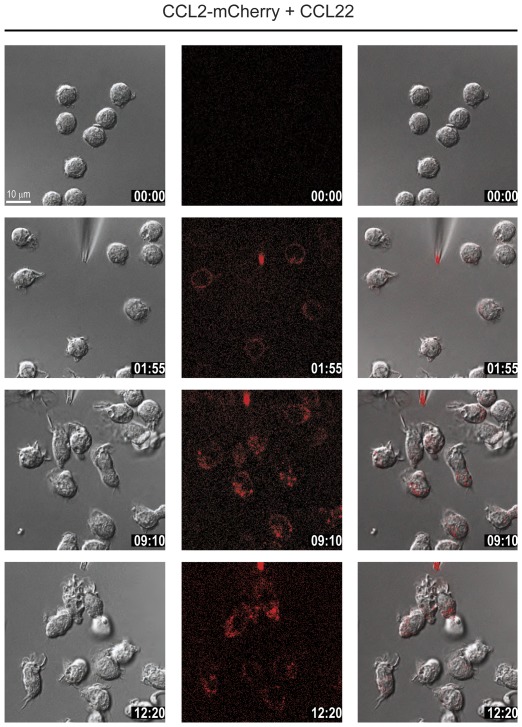
CCL2 uptake is D6-independent. Selected frames from time-lapse video microscopy (Movie S5) of migrating monocytes recorded at 37°C. The micropipette was dispending 100 nM CCL2-mCherry and 10 µM of the D6 ligand CCL22 with constant backpressure. Left panels phase images, middle panels CCL2-mCherry (ex 594 nm/em 620–680 nm), and right overlay. Time stamps are indicated in mm∶ss.

**Figure 5:**
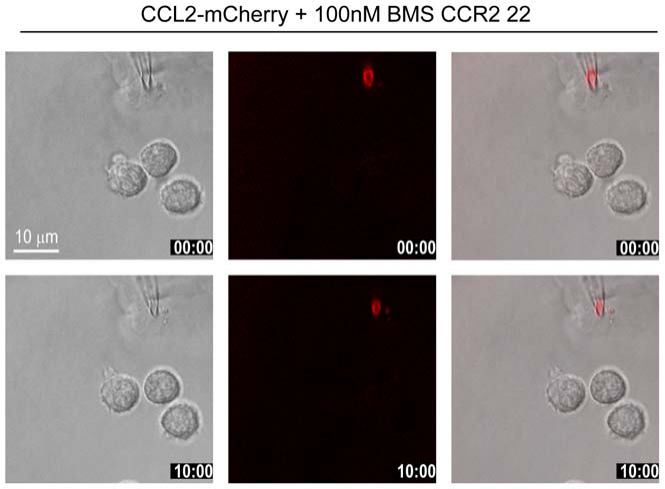
CCL2-mCherry uptake is blocked in the presence of an CCR2 antagonist. Selected frames from time-lapse video microscopy (Movie S6) of human monocytes recorded at 37°C in the presence of 100 nM of the CCR2 antagonist BMS CCR2 22. The micropipette was dispending 100 nM CCL2-mCherry with constant backpressure. Left panels phase images, middle panels CCL2-mCherry (ex 594 nm/em 620–680 nm), and right overlay. Time stamps are indicated in mm∶ss.

A second possibility of chemokine uptake could be mediated by scavenger receptors. Among the known mammalian chemokine scavenger receptors only D6 and DARC bind CCL2. The latter, however, is not expressed on monocytes [30]. D6 is a promiscuous receptor which binds multiple chemokines including CCL2 and CCL22, but not CCL20. To test the possible involvement of the scavenger receptor in chemokine uptake we used a ligand binding competition assay. The competition assay was chosen because antibodies that efficiently block the scavenging activity of D6 are not available. D6 binds CCL22 with slightly higher affinity than CCL2 [31]. Boyden chamber assays indicate that monocytes migrate in response to both chemokines although with different efficacies, CCL2 being more potent [32], [33]. In the time-lapse studies of chemotaxis shown in figure 4 and movie S5 the dispensing pipette was filled with 100 nM CCL2-mCherry in the presence of two order of magnitude larger concentration of CCL22 (10 µM). Under these conditions binding of CCL2 to D6 should be efficiently competed by CCL22, thus essentially blocking uptake of CCL2-mCherry. Figure 4 and movie S5 demonstrate that monocytes rapidly polarize and migrate towards the source of the chemoattractant. The marked internalization of CCL2-mCherry observed during migration was comparable in the presence or in the absence of CCL22, strongly arguing that uptake is mediated by CCR2 and not by D6. Since chemokine uptake is not mediated by pinocytosis or scavenger receptors it can be concluded that during monocyte migration CCR2 internalizes and deploys its cargo into lysosomes. This conclusion is also supported by the observation that uptake of CCL2-mCherry (100 nM) in migrating monocytes was markedly suppressed when administered together with 10 µM untagged wild-type CCL2 (not shown). By contrast, blocking CCL2 binding to CCR2 with a specific low molecular weight antagonist (BMS CCR2 22 [34]) fully inhibited migration and abrogated CCL2-mCherry uptake (Fig. 5 and movie S6). The observations further corroborate that endocytosis of the tagged chemokine is mediated by CCR2.

### Reversal of the chemotactic gradient switches monocyte polarity

**Figure 6:**
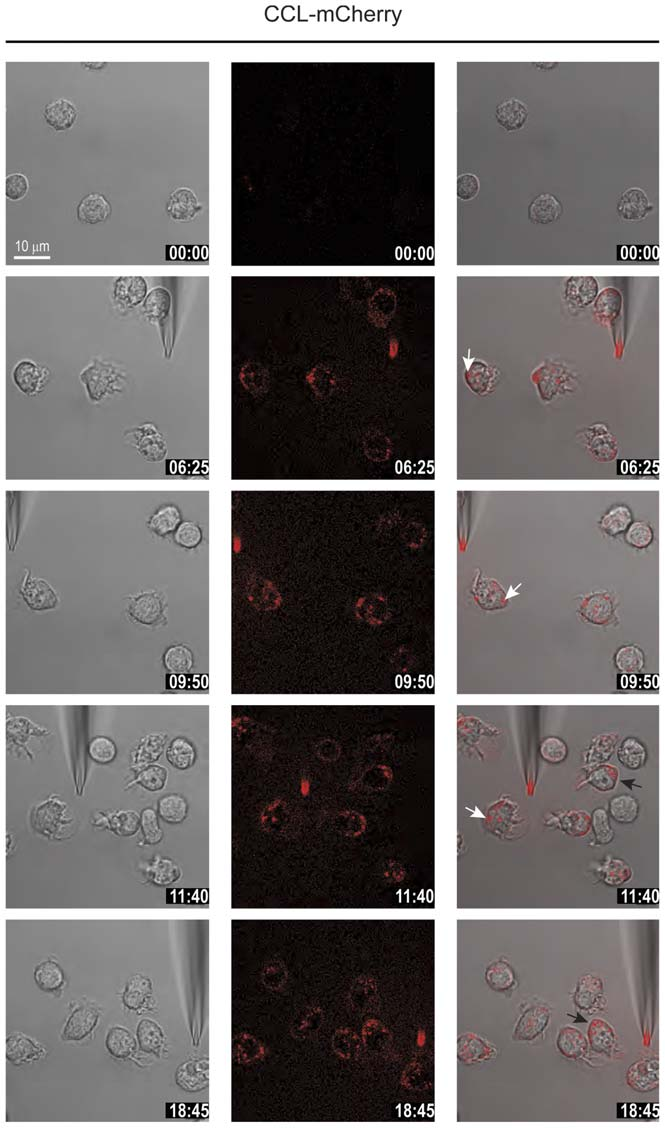
CCL2-mCherry uptake continues after reversal of polarization. Selected frames from time-lapse video microscopy (Movie S7) of migrating human monocytes recorded at 37°C. The micropipette was dispending 100 nM CCL2-mCherry with constant backpressure. The position of the needle was changed several times during recording. Left panels phase images, middle panels CCL2-mCherry (ex 594 nm/em 620–680 nm), and right overlay. Arrows indicate the localization of internalized CCL2-mCherry in two representative cells upon reverting their axis of polarization. Time stamps are indicated in mm∶ss.

We have previously shown that chemotaxis-mediating receptors remain equally distributed over the entire plasma membrane and that monocytes can rapidly reverse their polarization axis when the dispensing pipette is shifted from the front to the rear of the cell [25]. With the fluorescent chemokine in hand we were interested to test if chemokine uptake continues upon reversal of the gradient. Movie S7 (and Fig. 6) demonstrate that monocytes rapidly reverse their polarization axis when the pipette is moved to the opposite end of a polarized cell. Chemokine uptake continues and is accompanied by the relocation of the chemokine-loaded endosomes to the rear of the newly re-polarized cell. The movie also shows that the process can be repeated several times with the same outcome. This observation suggests that receptors internalize the bound fluorescent chemokine. While the cargo is deployed to endosomes for degradation the unloaded receptors recycle to the cell surface to maintain the responsiveness of the cell towards the attractant. In line with this we observed that after removal of the pipette from the surrounding medium the internalized chemokine is fully degraded within 15–20 min, but the cells remain responsive to a subsequent stimulation (movie S3). The observations indicate that chemokine receptors during migration consume the attractant from the medium acting as scavenging receptors.

### G-protein coupling is not required for chemokine uptake

**Figure 7:**
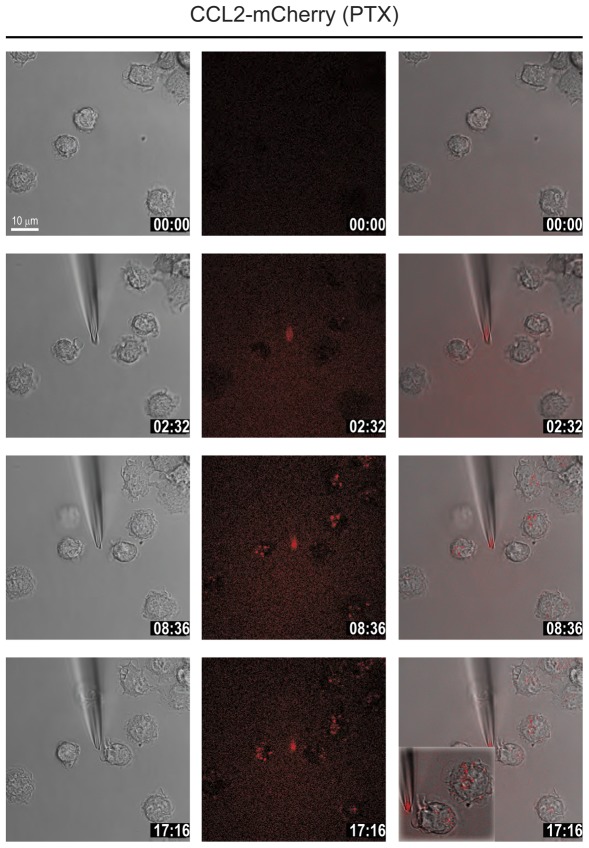
Pertussis toxin does not inhibit CCL2 uptake. Selected frames from time-lapse video microscopy (Movie S8) of migrating human monocytes recorded at 37°C. Monocytes were pretreated with 2 µg/ml *Bordetella* Pertussis toxin (PTX) for 2 h. The micropipette was dispending 100 nM CCL2-mCherry with constant backpressure. Left panels phase images, middle panels CCL2-mCherry (ex 594 nm/em 620–680 nm), and right overlay. Inset of bottom right panel, zoomed presentation of the same cells showing randomly localized internalized CCL2-mCherry. Time stamps are indicated in mm∶ss.

Chemokine receptors in general couple to pertussis toxin sensitive G_i_ proteins and therefore their functional responses are inhibited following treatment of cells with the toxin [1]. We preincubated monocytes with pertussis toxin to assess their capacity to respond to CCL2-mCherry in time-lapse studies. According to the notion that polarization depends on receptor-induced signaling toxin-treated cells did not polarize upon exposure to the fluorescent chemokine. Nevertheless, the cells were vibrant, actively forming filopodia- and lammelipodia-like protrusions, presumably triggered by pertussis toxin-independent mechanisms. Although they did not show any tendency to migrate in whichever direction, pertussis toxin treated monocytes internalized the CCL2-mCherry (Fig. 7 and movie S8). The fluorescence intensity of the endosomes increased over time indicating that the receptors continuously internalized the chemokine, which accumulated randomly within the cells, consistent with the non-polarized morphology. This finding suggests that receptor internalization is independent of cell migration, but intracellular distribution of chemokine loaded requires polarization.

### Phosphoinositide 3-kinase activity is dispensable for cell migration

**Figure 8:**
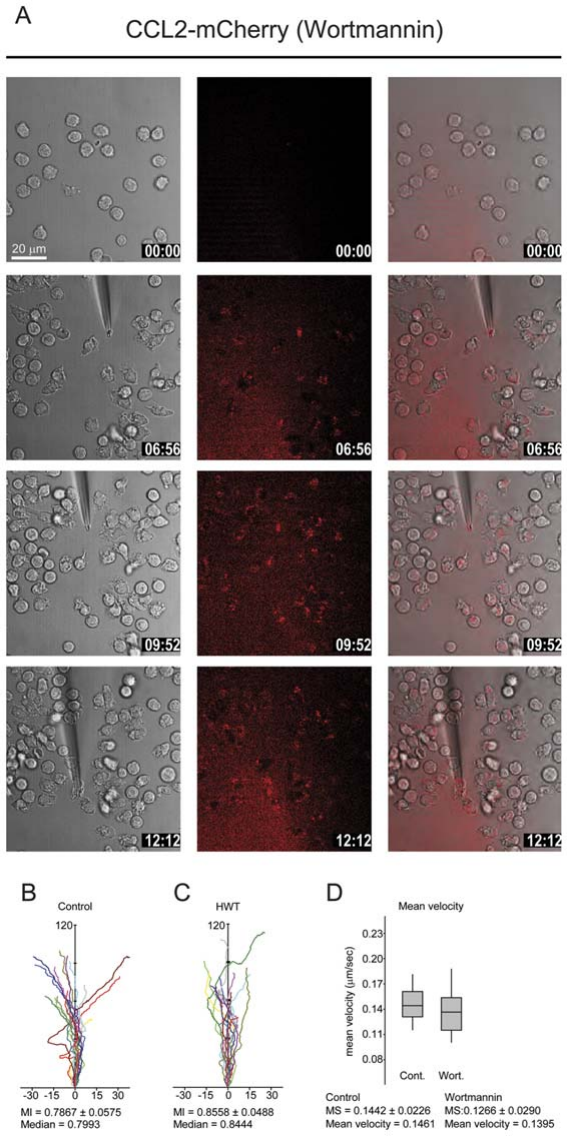
Inhibition of PI 3-kinase does not affect CCL2 uptake and monocyte migration. (A) Selected frames from time-lapse video microscopy (Movie S9) of migrating human monocytes recorded at 37°C. Monocytes were pretreated with 100 nM wortmannin for 30 min. The micropipette was dispending 100 nM CCL2-mCherry with constant backpressure. Gray (left column) corresponding phase image. Left panels phase images, middle panels CCL2-mCherry (ex 594 nm/em 620–680 nm), and right overlay. Time stamps are indicated in mm∶ss. (B and C) Analysis of directionality and velocity of monocytes: effect of PI 3-kinase inhibition. Tracks of monocytes (recorded as in Fig. 1 or Fig. 8A) either untreated (B) or pretreated with 100 nM wortmannin (C) were analyzed and plotted aligning their average directional vector with the y-axis (distance in µm). (D) Mean velocity of tracks shown in (B) and (C) were calculated using Metamorph software and median speed (MS) values are reported. Data from two independent observations, n = 16 (A) and n = 15 (B). MI = migratory index is the distance between the starting point and the end point divided by the total migrated distance.

The role of phosphoinositide 3-kinase (PI 3-kinase) in chemokine receptor trafficking and internalization has been debated. Inhibition of PI 3-kinase reduces the transferrin receptor expression and cycling and perturbs endosome fusion [35]. It was also shown that protein kinase activity of PI 3-kinase is required for the endocytosis of the β-adrenegic receptor [36]. However, for T lymphocytes it was reported that PI 3-kinase activity is not required for chemokine receptor internalization [37]. Thus, these reports do not support a consistent role of PI 3-kinase in receptor trafficking. We therefore investigated the potential role of PI 3-kinase in chemokine uptake during monocyte chemotaxis. Cells were pretreated for 20 min with 100 nM of the potent irreversible PI 3-kinase inhibitor wortmannin [38]. In previous studies and parallel experiments it was confirmed that this experimental regime is sufficient to fully abrogate PI 3-kinase activity (not shown). The cells were then exposed to CCL2-mCherry as before. Movie S9 and figure 8A show that wortmannin treated monocytes polarize and migrate towards the dispensing pipette similarly as untreated cells. Moreover the cells can rapidly flip their polarization axis following displacement of the pipette. During migration monocytes internalized continuously CCL2-mCherry, which accumulated in endosomes, that preferentially localized at the posterior end of the cells. Thus, inhibition of PI 3-kinase does not impair chemokine uptake and accumulation in large endosomes.

Inhibition of PI 3-kinase resulted in a minor change in the migratory index (MI). The MI is indicative for the efficiency of the cell movement representing the straightness of a migratory trace, calculated as the quotient of the shortest distance between the starting and the end point of a trace divided by the actual trace length (Fig. 8B and C). Similarly the velocity of the monocytes migration was not affected when PI 3-kinases activity was abrogated (Fig. 8D). The finding is consistent with previous data which unveiled that PI-3-kinase activity is dispensable for the migration of neutrophils, the monocytic cell line THP-1 and the amoeba *Dictyostelium* [25], [39]–[41].

## Discussion

In this study we investigated CCR2-mediated migration in primary human monocytes using the fluorescent receptor agonist CCL2-mCherry. Direct visualization of the endogenous CCR2 is not possible in primary cells. However, given that CCR2 is the only receptor for CCL2 expressed on monocytes, tracking the fluorescence of CCL2-mCherry allows to deduce conclusions on receptor-dependent cellular responses. Directional migration requires continuous signaling from receptors localized at the leading edge. This view is supported by the observation that upon reversal of the chemotactic cue monocytes rapidly switch their polarity (Fig. 6 and Movie S7 [25]) and orient the leading edge towards the new location of the chemokine source. Hence, receptors must be ubiquitously localized on the cell surface of migrating cells to be able to respond to moving attracting cues. CCR2-dependent migration of human monocytes towards the source dispensing CCL2 is accompanied by receptor-mediated ligand uptake. However, CCR2 internalization appears not to reduce the responsiveness of the cells to continuously migrate towards CCL2, suggesting that the receptors are rapidly recycled or that sufficient non-internalized receptors are available at the cell surface to maintain the responsiveness. In one study it was estimated that human monocytes express at their surface about 13'000 binding sites for CCL2 with relatively low affinity (K_d_ 25 nM) for the agonist [42]. However, other reports indicate a lower number (1200–1700) of high affinity (K_d_ 1–2 nM) binding sites for CCL2 [43], [44] and the high affinity binding of CCL2 on CCR2 was recently confirmed [32], [45], supporting the estimation of the lower receptor numbers. From our time-lapse video images we measured an average diameter of resting monocytes (e.g. Fig. 2A, movie S1) of 8.8732 µm (n = 28, from 14 cells measured on 2 perpendicular axes). Assuming a spherical shape of resting cells the calculated average surface area is ∼250 µm^2^, with approximately 5–10 receptor molecules per µm^2^. Thus, the number of CCR2 molecules facing the chemokine source is limited. Because CCL2 is most probably endocytosed as cargo bound to CCR2 and agonist uptake continues throughout migration, we conclude that receptor internalization must be compensated by rapid recycling. It is conceivable that the average number of receptors expressed at the monocyte surface drops upon continuous stimulation. However, the cells must be able to express sufficient receptors to maintain cell polarity and to mediate directional migration. Fig. 3 and 4 (Movies 4 and 5) show that alternative routes of chemokine uptake, such as pinocytosis or scavenger receptor-mediated, can be excluded and that blocking CCR2 abolishes CCL2-mChery internalization (Fig. 5 and Movie S6). Taken together our observations indicate that CCL2 is specifically taken up by CCR2 and that a stimulus-responsive fraction of the receptors must be expressed at the cell surface, supporting the conclusion that CCR2 cycles between the plasma membrane and endosomal compartments of monocytes, thereby consuming its ligand from the surrounding medium during migration.

It was previously shown that CCR2 expressed on monocytes can down regulate local chemokine levels *in vivo* [46]. Using CCL2 concentrations found in bronco-alveolar lavage fluids (600 pg/ml or ∼70 pM) *in vitro* measured CCR2-dependent consumption of CCL2 was reported as 450 pg/ml/100'000 cells in 24 h. Accordingly, monocytes would take up approx. 0.36 *10^−21^ moles/min equivalent to 1 molecule/receptor in 7–8 min. The determination is probably an underestimate of the maximum capacity due to the low concentration of chemokine offered in the incubation medium (∼70 pM) with respect to the K_d_ (1–2 nM) of CCR2 for CCL2 [46]. In the present study CCL2 was dispensed at 100 nM from the tip of micropipette. Taking into account the rapid dilution of the chemokine in the medium which reduces the concentration in a crude approximation by 50% every 20–30 µm (not shown), determinations of the chemoattractant concentration at the leading edge remain vague, due to the non-linear flow rate imposed by the narrow orifice of the pipette and the free diffusion of the ligand in the medium [25]. Nevertheless, monocyte migration can be induced with a wide range of CCL2 concentrations becoming effective at ∼30 nM (and tested up to 10 µM). No migration is observed below 10 nM CCL2 or with buffer alone. Thus, in the present experiments monocytes were exposed to local concentrations which were close to or significantly above K_d_. At these elevated concentrations of agonist receptor cycling is probably much higher than reported in [46] explaining the continuous marked uptake of CCL2-mCherry during chemotaxis.

In general chemokine receptors when exposed *in vitro* to their cognate chemokines are rapidly desensitized and become refractory to a subsequent stimulation [3]. Nevertheless, full receptor desensitization usually requires elevated ligand concentrations, i.e. 10–100 fold K_d_, as demonstrated by sequential stimulation of calcium mobilization [14], [47], [48]. Similarly, bulk stimulation with CCL2 induces the gradual internalization of CCR2, starting to be observed already at 0.1 nM CCL2, i.e. of one order of magnitude below K_d_, being prominent around K_d_ and most efficient at 100 fold K_d_ [20], [22]. Following CCL2-mediated down regulation, CCR2 surface expression is slowly restored reaching 100% and 70% after 4 h *in vivo* and *in vitro*, respectively [20]. Nevertheless, the newly expressed receptors were reported to be unable to stimulate migratory responses for up to 8 h, an observation that is in contrast with our findings [20]. First, our observations support the conclusion that receptors undergo several rounds of cycling between the plasma membrane and endosomes to maintain sustained responsiveness of the cells (e.g. for up to 20 min Movie S1). Second, monocytes stimulated for 15 min preserve their ability to respond immediately when re-exposed to CCL2 (Movie S3). The data suggest a qualitatively different effect on CCR2 when monocytes are stimulated in bulk, i.e. in suspension, or by chemotactic gradients while adhering to a matrix. Importantly, *in vivo* monocytes migrate being adherent to cell surfaces or matrix proteins rather than being in suspension, suggesting that cell-cell or cell-matrix interaction are required to observe the physiological behavior of CCR2. Bulk stimulation of monocytes with CCL2 leads to the rapid internalization of CCR2 passing through early and late endosomes before being degraded in lysosomes [11]. Tracking of the endocytic route in primary cells requires fixing and staining for endosomal markers. Such experiments are difficult to perform with the time-lapse video microscopy settings used to study human monocytes, where only few non-firmly adherent cells in close vicinity of the dispensing pipette migrate in the gradient. Transduction of freshly isolated monocytes with fluorescent-protein tagged endocytic markers is not recommended as the cells require over 24 h before the gene of interest reaches sufficient expression levels. During this time cultured monocytes lose their phenotype differentiating into macrophage-like cells with markedly different migratory properties. Tracking CCR2 could eventually be possible when monocytes from genetically engineered mice expressing fluorescent endosomal markers become available.

It is well established that pertussis toxin treatment abolishes monocyte migration (Fig. 5), but has no effect on arrestin recruitment and CCR2 internalization [3], [49], [50]. Conversely, CCR2 internalization can be dispensable for cell migration [22]. Mutation of serine to alanine residues in the C-terminus prevents CCR2 phosphorylation and abolishes receptor internalization which is mediated by a combination of clathrin-dependent and -independent pathways [7], [11], [19], [22]. Interestingly, at elevated CCL2 concentrations cells expressing a phosphorylation-deficient mutant CCR2 appear to be less efficient to migrate in Boyden chamber assays [22]. The finding is consistent with the conclusion that recycling (and cargo deployment) is not required *per se*, but might be needed to maintain the responsiveness at higher agonist concentrations. Our observations that CCL2-mCherry uptake persists in pertussis toxin-treated monocytes is in agreement with these previous reports demonstrating that toxin treatment abolishes migration, but does not affect CCR2 internalization. Furthermore we show that monocyte polarization and migration, as well as chemokine uptake and endosome fusion, are not attenuated by inhibition of PI-3kinase. The observation indicates that in migrating cells treated with wortmannin CCR2 can cycle between the surface and endosomal compartments. Wortmannin is known as potent irreversible pan-PI3K inhibitor, suggesting that PI 3-kinase activity is dispensable for signal transduction pathways regulating ligand uptake and endosme fusion. The finding is in agreement with the previous reported observations made in THP-1 cells [25]. Given the constrains discussed above on the necessity of receptor trafficking during cell migration it is reasonable to conclude that PI 3-kinase activity is also not required for CCR2 recycling. Taken together the present data, also obtained by the indirect measurement of fluorescent CCL2-mCherry uptake, indicate that chemokine receptors expressed on leukocytes internalize during chemotaxis and can act as scavengers for their cognate chemokines. The observation adds to the complexity of chemokine receptor signal transduction on leukocyte. The mechanism allows the cells to proceed along an increasing gradient of chemoattractant without being desensitized. In addition, consumption of the attractant represents a means by which migrating cells can reduce the strength of a chemotactic gradient for subsequently arriving leukocytes.

## Supporting Information

Movie S1 **Migration of freshly isolated human monocytes stimulated with CCL2-mCherry.** Frames were taken at 5 sec interval with a 63× magnification. Cells were stimulated with 100 nM CCL2-mCherry. Red fluorescence derives from the recombinant chemokine mCherry-tagged (ex 594 nm excitation/620-680 nm emission), phase images were taken with DIC settings. Overlay of phase and fluorescence is shown.
(AVI)

Movie S2 **Migration of freshly isolated human monocytes stimulated with CCL2-mCherry.** Confocal stacks (7) were taken at 15 sec interval with a 100× magnification. Cells were stimulated with 100 nM CCL2-mCherry. The surface of endosomes (blue) was calculated from the recorded red fluorescence CCL2- mCherry-tagged (ex 594 nm excitation/620–680 nm emission) using Imaris software, phase images were taken with DIC settings. Overlay of calculated surfaces and phase is shown.
(AVI)

Movie S3 **Migration of freshly isolated human monocytes stimulated with CCL2-mCherry.** Frames were taken at 5 sec interval with a 63× magnification. Cells were stimulated with 100 nM CCL2-mCherry. The dispensing micropipette was removed after 15 min and reinserted after 30 min. Red fluorescence derives from the recombinant chemokine mCherry-tagged (ex 594 nm excitation/620–680 nm emission), phase images were taken with DIC settings. Overlay of phase and fluorescence is shown.
(AVI)

Movie S4 **Monocyte migration stimulated in the presence of CCL2-mCherry and CCL20-EYP.** Frames were taken at 5 sec interval with a 63× magnification. The stimuli were released from a micropipette dispensing a solution containing 1 µM CCL2-mCherry and 1 µM CCL20-EYFP. The emission of CCL2-mCherry (594 nm excitation/620–680 nm emission) and CCL20-YFPP (488 nm exitation 500–520 nm emission) was recorded simultaneously. Phase images were taken with DIC settings. Overlay of phase and fluorescence is shown.
(AVI)

Movie S5 **Monocyte migration stimulated in the presence of CCL2-mCherry and CCL22.** Frames were taken at 5 sec interval with a 63× magnification. The stimuli were released from a micropipette dispensing a solution containing 100 nM CCL2-mCherry and 10 µM CCL22. The emission of CCL2-mCherry (594 nm excitation/620–680 nm emission) was overlaid with phase images taken with DIC settings.
(AVI)

Movie S6 **CCL2-mCherry uptake is fully inhibited in the presence of 100 nM of the CCR2 antagonist BMS CCR2 22.** Monocytes were pretreated with the antagonist for 10 min before insertion of the micropipette dispending 100 nM CCL2-mCherry. Frames were taken at 15 sec interval with a 100× magnification. Red fluorescence derives from the recombinant chemokine mCherry-tagged (ex 594 nm excitation/620–680 nm emission), phase images were taken with DIC settings. Overlay of phase and fluorescence is shown
(AVI)

Movie S7 **Relocation of the attractant source has no effect on CCL2-mCherry uptake.** Frames were taken at 5 sec interval with a 63× magnification. Cells were stimulated with 100 nM CCL2-mCherry. The position of the micropipette dispensing 100 nM CCL2-mCherry was changed several times during recording. The emission of CCL2-mCherry (594 nm excitation/620–680 nm emission) was overlaid with phase images taken with DIC settings.
(AVI)

Movie S8 **CCL2-mCherry uptake is not affected by pertusiss toxin treatment.** Monocytes were pretreated with 2 µg/ml *Bordetella* Pertussis toxin for 2 h. The micropipette was dispending 100 nM CCL2-mCherry. Frames were taken at 5 sec interval with a 63× magnification. Red fluorescence derives from the recombinant chemokine mCherry-tagged (ex 594 nm excitation/620–680 nm emission), phase images were taken with DIC settings. Overlay of phase and fluorescence is shown.
(AVI)

Movie S9 **Inhibition of PI 3-kinase does not affect CCL2 uptake and monocyte migration.** Monocytes were pretreated with 100 nM wortmannin for 30 min. The micropipette was dispending 100 nM CCL2-mCherry. Red fluorescence derives from the recombinant chemokine mCherry-tagged (ex 594 nm excitation/620–680 nm emission), phase images were taken with DIC settings. Overlay of phase and fluorescence is shown.
(AVI)

## References

[1] Thelen M, Stein JV (2008). How chemokines invite leukocytes to dance.. Nat Immunol.

[2] Murphy PM, Baggiolini M, Charo IF, Hebert CA, Horuk R (2000). International union of pharmacology. XXII. Nomenclature for chemokine receptors.. Pharmacol Rev.

[3] Thelen M (2001). Dancing to the tune of chemokines.. Nat Immunol.

[4] Pollok-Kopp B, Schwarze K, Baradari VK, Oppermann M (2003). Analysis of ligand-stimulated CC Chemokine receptor 5 (CCR5) phosphorylation in intact cells using phosphosite-specific antibodies.. J Biol Chem.

[5] Zhang N, Yang D, Dong H, Chen Q, Dimitrova DI (2006). Adenosine A2a receptors induce heterologous desensitization of chemokine receptors.. Blood.

[6] Oppermann M, Mack M, Proudfoot AE, Olbrich H (1999). Differential effects of CC chemokines on CC chemokine receptor 5 (CCR5) phosphorylation and identification of phosphorylation sites on the CCR5 carboxyl terminus.. J Biol Chem.

[7] Neel NF, Schutyser E, Sai J, Fan GH, Richmond A (2005). Chemokine receptor internalization and intracellular trafficking.. Cytokine Growth Factor Rev.

[8] Tobin AB, Butcher AJ, Kong KC (2008). Location, location, location…site-specific GPCR phosphorylation offers a mechanism for cell-type-specific signalling.. Trends Pharmacol Sci.

[9] Signoret N, Hewlett L, Wavre S, Pelchen-Matthews A, Oppermann M (2005). Agonist-induced endocytosis of CC chemokine receptor 5 is clathrin dependent.. Mol Biol Cell.

[10] Venkatesan S, Rose JJ, Lodge R, Murphy PM, Foley JF (2003). Distinct mechanisms of agonist-induced endocytosis for human chemokine receptors CCR5 and CXCR4.. Mol Biol Cell.

[11] Garcia Lopez MA, Aguado MA, Lamaze C, Martinez A, Fischer T (2009). Inhibition of dynamin prevents CCL2-mediated endocytosis of CCR2 and activation of ERK1/2.. Cell Signal.

[12] Borroni EM, Mantovani A, Locati M, Bonecchi R (2010). Chemokine receptors intracellular trafficking.. Pharmacol Ther.

[13] Moser B, Wolf M, Walz A, Loetscher P (2004). Chemokines: multiple levels of leukocyte migration control.. Trends Immunol.

[14] Charo IF, Myers SJ, Herman A, Franci C, Connolly AJ (1994). Molecular cloning and functional expression of two monocyte chemoattractant protein 1 receptors reveals alternative splicing of the carboxyl-terminal tails.. Proc Natl Acad Sci USA.

[15] Franci C, Wong LM, Van Damme J, Proost P, Charo IF (1995). Monocyte chemoattractant protein-3, but not monocyte chemoattractant protein-2, is a functional ligand of the human monocyte chemoattractant protein-1 receptor.. J Immunol.

[16] Godiska R, Chantry D, Raport CJ, Schweickart VL, Trong HL (1997). Monocyte chemotactic protein-4: tissue-specific expression and signaling through CC chemokine receptor-2.. J Leukoc Biol.

[17] Dubois PM, Palmer D, Webb ML, Ledbetter JA, Shapiro RA (1996). Early signal transduction by the receptor to the chemokine monocyte chemotactic protein-1 in a murine T cell hybrid.. J Immunol.

[18] Sozzani S, Zhou D, Locati M, Rieppi M, Proost P (1994). Receptors and transduction pathways for monocyte chemotactic protein-2 and monocyte chemotactic protein-3: Similarities and differences with MCP-1.. J Immunol.

[19] Berchiche YA, Gravel S, Pelletier ME, St-Onge G, Heveker N (2011). Different effects of the different natural CC chemokine receptor 2b ligands on beta-arrestin recruitment, Galphai signaling, and receptor internalization.. Mol Pharmacol.

[20] Handel TM, Johnson Z, Rodrigues DH, Dos Santos AC, Cirillo R (2008). An engineered monomer of CCL2 has anti-inflammatory properties emphasizing the importance of oligomerization for chemokine activity in vivo.. J Leukoc Biol.

[21] Franci C, Gosling J, Tsou CL, Coughlin SR, Charo IF (1996). Phosphorylation by a G protein-coupled kinase inhibits signaling and promotes internalization of the monocyte chemoattractant protein-1 receptor. Critical role of carboxyl-tail serines/threonines in receptor function.. J Immunol.

[22] Arai H, Monteclaro FS, Tsou CL, Franci C, Charo IF (1997). Dissociation of chemotaxis from agonist-induced receptor internalization in a lymphocyte cell line transfected with CCR2B - Evidence that directed migration does not require rapid modulation of signaling at the receptor level.. J Biol Chem.

[23] Nieto M, Frade JMR, Sancho D, Mellado M, Martinez C (1997). Polarization of chemokine receptors to the leading edge during lymphocyte chemotaxis.. J Exp Med.

[24] Servant G, Weiner OD, Neptune ER, Sedat JW, Bourne HR (1999). Dynamics of a chemoattractant receptor in living neutrophils during chemotaxis.. Mol Biol Cell.

[25] Volpe S, Thelen S, Pertel T, Lohse MJ, Thelen M (2010). Polarization of migrating monocytic cells is independent of PI 3-kinase activity.. PLoS ONE.

[26] Shaner NC, Campbell RE, Steinbach PA, Giepmans BN, Palmer AE (2004). Improved monomeric red, orange and yellow fluorescent proteins derived from Discosoma sp. red fluorescent protein.. Nat Biotechnol.

[27] Garotta G, Thelen M, Delia D, Kamber M, Baggiolini M (1991). GM-1, a clone of the monoblastic phagocyte U937 that expresses a large respiratory burst capacity upon activation with interferon-gamma.. J Leukocyte Biol.

[28] Shaner NC, Steinbach PA, Tsien RY (2005). A guide to choosing fluorescent proteins.. Nat Methods.

[29] Nibbs RJB, Wylie SM, Pragnell IB, Graham GJ (1997). Cloning and characterization of a novel murine b chemokine receptor, D6 - Comparison to three other related macrophage inflammatory protein-1a receptors, CCR-1, CCR-3, and CCR- 5.. J Biol Chem.

[30] Graham GJ (2009). D6 and the atypical chemokine receptor family: novel regulators of immune and inflammatory processes.. Eur J Immunol.

[31] Savino B, Borroni EM, Machado TN, Proost P, Struyf S (2009). Recognition versus adaptive upregulation and degradation of CC chemokines by the chemokine decoy receptor D6 are determined by their N-terminal sequence.. J Biol Chem.

[32] Ogilvie P, Bardi G, Clark-Lewis I, Baggiolini M, Uguccioni M (2001). Eotaxin is a natural antagonist for CCR2 and an agonist for CCR5.. Blood.

[33] Godiska R, Chantry D, Raport CJ, Sozzani S, Allavena P (1997). Human macrophage-derived chemokine (MDC), a novel chemoattractant for monocytes, monocyte-derived dendritic cells, and natural killer cells.. J Exp Med.

[34] Cherney RJ, Mo R, Meyer DT, Nelson DJ, Lo YC (2008). Discovery of disubstituted cyclohexanes as a new class of CC chemokine receptor 2 antagonists.. J Med Chem.

[35] Spiro DJ, Boll W, Kirchhausen T, Wessling-Resnick M (1996). Wortmannin alters the transferrin receptor endocytic pathway in vivo and in vitro.. Mol Biol Cell.

[36] Naga Prasad SV, Jayatilleke A, Madamanchi A, Rockman HA (2005). Protein kinase activity of phosphoinositide 3-kinase regulates beta-adrenergic receptor endocytosis.. Nat Cell Biol.

[37] Sauty A, Colvin RA, Wagner L, Rochat S, Spertini F (2001). CXCR3 internalization following T cell-endothelial cell contact: preferential role of IFN-inducible T cell alpha chemoattractant (CXCL11).. J Immunol.

[38] Thelen M, Wymann MP, Langen H (1994). Wortmannin binds specifically to 1-phosphatidylinositol 3-kinase while inhibiting guanine nucleotide-binding protein- coupled receptor signaling in neutrophil leukocytes.. Proc Natl Acad Sci USA.

[39] Turner SJ, Domin J, Waterfield MD, Ward SG, Westwick J (1998). The CC chemokine monocyte chemotactic peptide-1 activates both the class I p85/p110 phosphatidylinositol 3-kinase and the class II PI3K- C2alpha.. J Biol Chem.

[40] Hoeller O, Kay RR (2007). Chemotaxis in the absence of PIP3 gradients.. Curr Biol.

[41] Ferguson GJ, Milne L, Kulkarni S, Sasaki T, Walker S (2007). PI(3)Kgamma has an important context-dependent role in neutrophil chemokinesis.. Nat Cell Biol.

[42] Wang JM, Hishinuma A, Oppenheim JJ, Matsushima K (1993). Studies of binding and internalization of human recombinant monocyte chemotactic and activating factor (MCAF) by monocytic cells.. Cytokine.

[43] Yoshimura T, Leonard EJ (1990). Identification of high affinity receptors for human monocyte chemoattractant protein-1 on human monocytes.. J Immunol.

[44] Valente AJ, Rozek MM, Schwartz CJ, Graves DT (1991). Characterization of monocyte chemotactic protein-1 binding to human monocytes.. Biochem Biophys Res Commun.

[45] Sohy D, Parmentier M, Springael JY (2007). Allosteric trans-inhibition by specific antagonists in CCR2/CXCR4 heterodimers.. J Biol Chem.

[46] Maus UA, Wellmann S, Hampl C, Kuziel WA, Srivastava M (2005). CCR2-positive monocytes recruited to inflamed lungs downregulate local CCL2 chemokine levels.. Am J Physiol Lung Cell Mol Physiol.

[47] Gong J-H, Clark-Lewis I (1995). Antagonists of monocyte chemoattractant protein 1 identified by modification of functionally critical NH_2_-terminal residues.. J Exp Med.

[48] Baggiolini M, Dewald B, Moser B (1994). Interleukin-8 and related chemotactic cytokines–CXC and CC chemokines.. Adv Immunol.

[49] Panzer U, Uguccioni M (2004). Prostaglandin E2 modulates the functional responsiveness of human monocytes to chemokines.. Eur J Immunol.

[50] Arai H, Tsou CL, Charo IF (1997). Chemotaxis in a lymphocyte cell line transfected with C- C chemokine receptor 2B: Evidence that directed migration is mediated by bgamma dimers released by activation of G_ai_- coupled receptors.. Proc Natl Acad Sci USA.

